# Fate of Ergot Alkaloids during Laboratory Scale Durum Processing and Pasta Production

**DOI:** 10.3390/toxins11040195

**Published:** 2019-03-31

**Authors:** Sheryl A. Tittlemier, Dainna Drul, Mike Roscoe, Dave Turnock, Dale Taylor, Bin Xiao Fu

**Affiliations:** Grain Research Laboratory, Canadian Grain Commission, 1404-303 Main Street, Winnipeg, MB R3C 3G8, Canada; dainna.drul@grainscanada.gc.ca (D.D.); mike.roscoe@grainscanada.gc.ca (M.R.); dave.turnock@grainscanada.gc.ca (D.T.); dale.taylor@grainscanada.gc.ca (D.T.); binxiao.fu@grainscanada.gc.ca (B.X.F.)

**Keywords:** mycotoxin, milling, bran, semolina, cooking, dietary exposure

## Abstract

The fate of ergot alkaloids during the milling of durum and subsequent production and cooking of pasta was examined. Durum samples containing varying amounts of ergot sclerotia (0.01–0.1% by mass) were milled, and all milling product was analyzed for 10 ergot alkaloids using liquid chromatography with tandem mass spectrometry. Spaghetti was prepared from the semolina obtained during milling. Ergocristine, ergocristinine, and ergotamine were the predominant ergot alkaloids observed in the milling fractions and spaghetti. Approximately 84% of the total ergot alkaloid mass of the whole grain durum resided in the milling product fractions associated with the outer kernel layers (bran, shorts, feeds). No consistent loss of ergot alkaloids was observed during the production or cooking of spaghetti. However, changes in the ratio of *R*- to *S*-enantiomers occurred during the milling and cooking of spaghetti. Products containing bran, shorts, and feeds, as well as cooked spaghetti, contained a higher proportion of the less biologically active *S*-enantiomers. The results of this study emphasize the need to monitor *R*- and *S*-enantiomers, and to consider food and feed products, as opposed to whole grain, when assessing any exposure of consumers to ergot alkaloids.

## 1. Introduction

Ergot alkaloids (EAs) are a group of mycotoxins produced during the fungal infection of cereals. In Canada, the fungus *Claviceps purpurea* causes ergot infection of rye, barley, oats, wheat, and durum [1]. During *C. purpurea* infection, healthy kernels are replaced by dark-colored sclerotia that contain high concentrations of various EAs [2,3,4]. The incidence of ergot infection of durum grown in Canada is variable amongst growing years, but appears to be increasing since the early 2000s [5]. This increase suggests that the potential for EAs occurring in durum, and durum-based food products, is also increasing. A number of EAs have been measured in Canadian cereals [5], as well as those grown in western [6] and eastern Europe [7], and Australia [3].

The EAs most commonly associated with cereal grains are amide-like derivatives of lysergic acid [8]. These compounds contain a stereogenic center, and can exist in *R*- (indicated by an “ine” suffix in their names) and *S*- (indicated by an “inine” suffix in their names) enantiomeric forms. The enantiomers can undergo reversible epimerization, which can reach equilibrium. Epimerization has been reported to occur in solution under alkaline conditions and lengthy periods of storage [9].

Consumption of ergot alkaloids has been long known to cause ergotism in both humans and livestock [10,11]. Symptoms of ergotism include gangrene, gastrointestinal effects, reduced lactation, as well as effects on the central nervous system.

Current practices to avoid or minimize exposure to ergot alkaloids in food and feed involve grain handling and milling procedures to exclude ergot sclerotia from entering food and feed channels. Tolerances are also used to regulate the amount of ergot sclerotia in grain. For example, the Canadian maximum level for ergot sclerotia in durum is 0.02% on a mass basis for the higher quality grades of No.1 Canada Western Amber Durum (CWAD) and No.2 CWAD [12], and the Codex Alimentarius Commission maximum level of ergot sclerotia in wheat is 0.05% [13].

Currently, no jurisdictions have set maximum levels (MLs) for ergot alkaloids in grain, food, or feed. However, regulatory agencies are exploring the use of MLs as a tool to manage health risk from exposure to EAs. The Canadian Food Inspection Agency (CFIA) consulted stakeholders on proposed limits for a number of contaminants, including ergot alkaloids, in livestock feeds [14]. The complexity of EA exposure and hazard assessments due to processing effects on EA concentrations, toxicity of individual EAs, and synergistic or antagonistic effects in EA mixtures, as well as variable susceptibilities due to age, sex, and physiological state, coupled with limited published scientific information in these areas, led the CFIA to revisit setting MLs for EAs in the future [15].

There are limited published studies on the fate of ergot alkaloids in food or feed products during the processing of grain. Most of the studies focus on rye-based food and feed. Fajardo et al. [16] investigated the fate of six EAs in milling products of red spring wheat. Flour containing EAs was also used to prepare pasta, Asian noodles, and bread. Overall, EAs were concentrated in the milling products associated with reduction streams, and some losses were noted in cooked noodles. However, a limitation of this work is the inclusion of only *R*-enantiomers in the EA analyses. Merkel et al. [17] studied the fate of twelve EAs in cookies baked with rye flour and subjected to in vitro digestion. The authors reported degradation of EAs during baking, as well as a shift from *R*- to *S*-enantiomers for all EAs. A feeding study conducted by Dänicke [18] included an assessment of the fate of twelve EAs in rye-based feed for laying hens, which had been processed under heat and pressure. Substantial epimerization of EAs was noted after processing, with an increase in concentration of *S*-enantiomers for all EAs.

The objective of this work was to take advantage of ongoing pasta quality and functionality studies, and examine the fate of EAs in milled durum, as well as during the processing and cooking of spaghetti. This study used durum, a cereal grain important in the production of pasta. The fate of EAs during the processing of durum has not been well studied. This study also monitored the fate of four *R*/*S* enantiomeric pairs, as well as two additional EA *R*-enantiomers for which the *S*-enantiomer was not readily available at the time of the study. The outcomes of this study are valuable for use in exposure assessments to ensure that consumers’ exposure to EAs in food, or feed, is accurately determined.

## 2. Results

### 2.1. Ergot Alkaloids in Whole Grain and Milling Products

Table 1 lists the EA concentrations measured in the comminuted whole grain and milling products for the six samples of CWAD containing varying amounts of ergot sclerotia. Concentrations are provided as the sum of 10 ergot alkaloid analyte concentrations. Milling yields from the two mill runs of 2 kg each are also listed in Table 1.

Total EA concentrations for whole grain and all milling products increased as the percentage of ergot sclerotia in the original durum sample increased. The correlation between the percentage of ergot sclerotia and total EA concentration was statistically significant for whole grain and all milling products (Pearson Product Moment Correlation, *p* < 0.0025), aside from bran (*p* < 0.111). The total EA concentration of bran increased from 1112 to 5695 μg/kg as the ergot sclerotia content increased from 0.01% to 0.1%, and reached a maximum of 5715 μg/kg at an ergot sclerotia content of 0.04%.

EAs were observed in all fractions from the five samples that contained ergot sclerotia. Only feeds and the first semolina fraction from the CWAD sample with no visible ergot sclerotia contained measurable EAs. Total EA concentrations in these fractions were low, ranging from 12 to 16 μg/kg. Total EA concentrations from the other samples were highest in bran, shorts, and feeds and the lowest was in the first semolina fraction. The concentrations in this semolina fraction were approximately 20–40× lower than the maximum measured in bran, shorts, or feeds.

**Figure 1:**
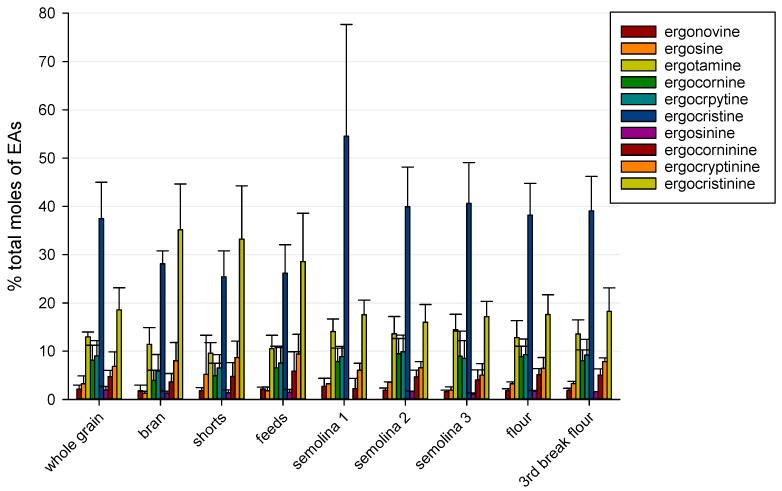
Mean ± standard deviation percentage of total moles of ergot alkaloids present in whole grain and milling products. Means and standard deviations were calculated from whole grain and milling products from the five durum samples with ergot sclerotia ranging from 0.01–0.1% by mass.

Ergocristine and ergocristinine, followed by ergotamine, were the most predominant EAs observed in whole grain and milling products. The mean percentage of total EAs on a molar (as opposed to mass-based concentration) basis for each EA in the five samples containing ergot sclerotia was determined; these are presented in Figure 1.

In bran, shorts, and feeds, ergocristinine was the predominant EA at 29–35% of total EAs. The molar percentage of ergocristinine in bran and shorts were significantly different from those of the semolina and flour fractions (*p* < 0.05). Ergocristine was predominant in whole grain and other milling products, ranging from 37–55% of total EAs. The mean molar percentage of ergocristine was significantly different between semolina 1 and bran, shorts, and feeds (*p* < 0.05). 

No other significant differences were noted amongst the other milling products. Ergotamine ranged from 10–14% of total EAs in whole grain and all milling products.

### 2.2. Ergot Alkaloids in Spaghetti

Table 2 lists the mean recoveries of individual EAs from fortified boiled spaghetti and cooking water. Mean recoveries for all EAs ranged from 93% to 113% from cooking water. Mean recoveries from boiled spaghetti were assessed only for the *R* enantiomers in order to preserve standard material, and because no difference in recoveries was observed for cooking water and whole grain [5]. Mean recoveries of the *R* enantiomer EAs from fortified boiled spaghetti ranged from 98% to 143%.

Table 3 lists concentrations measured in freshly extruded spaghetti, cooked spaghetti, and cooking water from the six samples of CWAD containing varying amounts of ergot sclerotia. The moisture contents of the spaghetti are also provided. The concentrations provided in Table 3 are on a fresh weight basis; that is, they are not normalized to a specific moisture content. The moisture content of cooked spaghetti was approximately twice that of the freshly extracted spaghetti.

Similar to whole grain, semolina, and flour fractions, ergocristine, ergocristinine, and ergotamine were the most predominant EAs observed in freshly extruded spaghetti. The mean percentage and standard deviation of total EAs on a molar basis for these three EAs in the five samples containing ergot sclerotia were 42 ± 8% for ergocristine, 19 ± 3% for ergocristinine, and 15 ± 5% for ergotamine. For cooked spaghetti and cooking water, ergocristinine was the predominant EA at 55 ± 8% and 73 ± 28% of the total EA molar content, respectively.

## 3. Discussion

### 3.1. Fate of Ergot Alkaloids in Milling Products and Spaghetti

**Figure 2:**
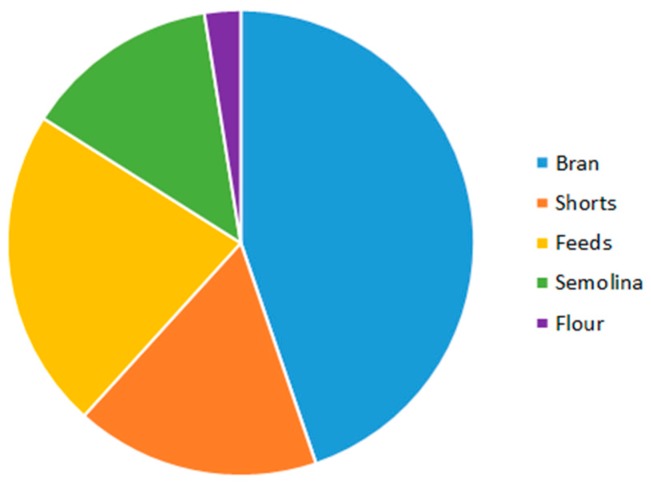
Distribution of total ergot alkaloids mass amongst durum milling products. Pie slices represent the mean fraction of total ergot alkaloid mass across the five durum samples with ergot sclerotia ranging from 0.01–0.1% by mass.

Most of the EAs associated with sclerotia were in the by-products of semolina milling, i.e., bran, shorts, and feeds from outer kernel layers. Higher amounts of EAs were associated with bran, shorts, feeds (Figure 2), which are milling products containing material from the outer kernel. These three milling products contained 84% of the total EA mass in the durum.

The presence of the EAs predominantly in these milling fractions is consistent with the observation by Franzmann et al. [19] that the amounts of EAs in rye flour increased with a higher amount of bran in the flour. Franzmann et al.’s work attributed this EA distribution on rye kernels to the coincidental contact and abrasion between sound rye and ergot sclerotia during ordinary grain handling and movement.

Even though EAs will be transferred to sound kernels from ergot sclerotia during movement, the fate of ergot sclerotia during the milling process will be the most important factor affecting EA content of milling products because the concentration of EAs in ergot sclerotia are orders of magnitude greater than in sound grain. Total EA concentrations in rye ergot sclerotia were approximately 300× greater than concentrations in rye that had been mixed with ground ergot sclerotia and subject to cleaning [19]. Mean concentrations of the 10 EAs included in the current study ranged from 500 to 1000 mg/kg in ergot sclerotia obtained from infected durum plants [4].

The predominance of EAs in the bran, shorts, and feeds milling products fractions is also consistent with the path taken by ergot sclerotia through a wheat milling procedure reported by Farjado et al. [16]. The aforementioned Farjado et al. [16] milled wheat containing various amounts of ergot sclerotia. They noted that the sclerotia congregated in the reduction system because they did not flake when passing through the break rolls. This led to approximately 75% of EAs present in the whole grain wheat residing in bran and shorts milling fractions in their study.

The fate of the individual EAs was also examined over the milling process. Interestingly, there were differences in the EA profiles amongst the milling products associated with the outer kernel layers (bran, shorts, and feeds) and endosperm (semolina, flour), in addition to the variation in concentration.

**Figure 3:**
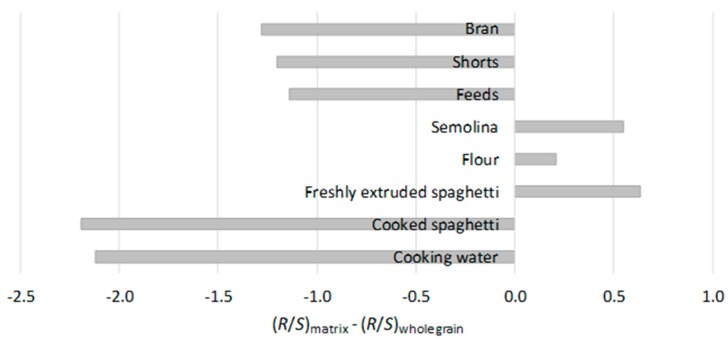
Difference in ratios of *R*-enantiomer to *S*-enantiomer concentrations between whole grain durum, milling products, and pasta matrices. The difference in ratios was calculated using mean *R*/*S* concentration ratios determined from the five groups containing 0.01–0.1% ergot sclerotia by mass.

These differences in EA profiles are illustrated in Figure 3. The ratio of *R*- to *S*-enantiomers was lower in milling products associated with the outer kernel layers as compared to whole grain, indicating a predominance of the *S*-enantiomers. The *R*-enantiomers were predominant in semolina and flour, and were present in these fractions to greater extent than in whole grain.

The work in this study does not directly address or examine the cause of the differences in occurrence amongst individual EAs. However, past research has investigated or noted the epimerization of EAs. Epimerization of EAs is reported to be promoted by exposure to light [20], therefore EAs in the outer kernel layers may be subject to more light and subsequent epimerization than EAs in the inner kernel layers. Even though heat also appears to facilitate epimerization [17,18], it is unlikely that the milling process used in this study promoted epimerization in the bran, shorts, and feeds fractions, as temperatures generated during the roller milling of wheat are around 35 °C [21].

**Figure 4:**
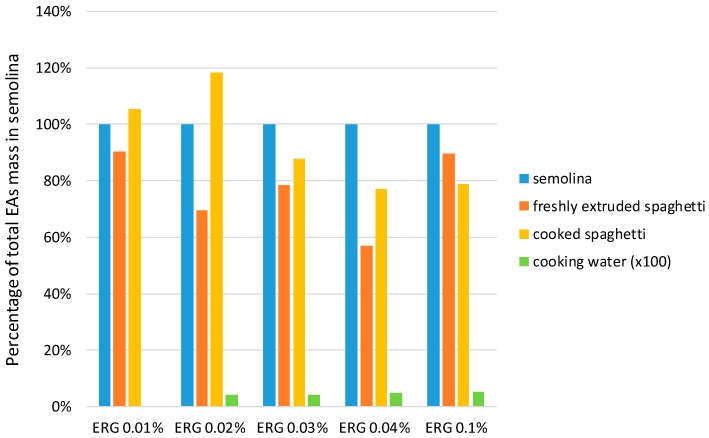
Amount of total ergot alkaloids in processed spaghetti and cooking water relative to semolina. The mean of duplicates is reported for freshly extruded spaghetti. Single analyses were performed for the other matrices.

The cooking of spaghetti did not appear to considerably affect the presence of EAs. No substantial losses of EAs were consistently evident after the preparation of spaghetti by extrusion, nor after boiling the spaghetti. Figure 4 provides a comparison of the amount of EAs in freshly extruded spaghetti, cooked spaghetti, and the cooking water, to semolina. The comparison is on the basis of EA mass, therefore the impact from varying moisture content of the products is avoided. Across the five durum samples with varying ergot sclerotia contents, freshly extruded spaghetti and cooked spaghetti samples contained 77 ± 14% and 93 ± 18% of the total EA content observed in semolina, respectively. Cooking water contained a negligible amount of EAs (0.04 ± 0.02%) of the total semolina EA content.

Overall, the results observed do not indicate a consistent and extensive loss of EAs during processing. While the amounts of EAs observed in the freshly extruded spaghetti seem to suggest some loss, the amounts of EAs observed in cooked spaghetti do not demonstrate similar losses. The apparently lower EA amounts measured in the freshly extruded spaghetti may reflect differences in the ability of the analytical method to extract and/or measure EAs in this matrix.

Merkel et al. [17] reported small losses of 2–30% in cookies due to degradation of EAs during baking. Dänicke noted an average loss of 11% for EAs in heat treated rye [18], but the changes in concentrations across the five chicken diets examined, ranged from a loss of 26%, to an increase of 15%. This inconsistency suggests that the heterogeneous nature of ergot contamination of whole grain may have contributed to the apparent loss of EAs in the heat treated rye.

Fajardo et al. [16] also reported losses of EAs during the cooking of Asian noodles and spaghetti made with wheat flour. However the analytical method used in that study only included the *R*-enantiomers as analytes. Any occurrence of *S*-enantiomers formed by epimerization during cooking would not be observed. Therefore changes in concentrations of EAs due to epimerization would appear instead as losses of EAs.

Even though no substantial losses of EAs were observed during the processing and cooking of spaghetti, the EA profile did change during cooking. As seen in Figure 3, the EA content of semolina and freshly extruded spaghetti is dominated by *R*-enantiomers, which changed to a predominance of *S*-enantiomers in cooked spaghetti and cooking water. The changes in the EA profile observed suggest that the heat of extrusion (45 °C) used to prepare the fresh spaghetti is not enough to promote epimerization, whereas the heat of boiling water can facilitate epimerization.

The enrichment of *S*-enantiomers after observed after cooking spaghetti is consistent with changes observed in other research. Merkel et al. reported the epimeric ratio shifted toward the *S*-enantiomer for all EAs in baked cookies [17]. Dänicke [18] also noted a consistent increase in the proportion of *S*-enantiomers (and concomitant decrease in *R*-enantiomers) in rye with varying ergot sclerotia content after heat treatment.

### 3.2. Implications of Ergot Alkaloid Fate in Milling Products and Spaghetti

The wider implications of this work relate to the distribution of EAs amongst the durum milling products and the epimerization of EAs observed. The association of EAs with the bran, shorts, and feeds fractions after durum milling will lower the exposure for populations consuming food products, such as pasta, made from semolina, as compared to whole grain durum. In turn, any incorporation of bran, shorts, and feeds fractions into animal feed will increase exposure of livestock, as compared to use of whole grain durum.

The epimerization observed from cooking and milling durum indicate that feed products and cooked pasta will contain a higher proportion of the less biologically active *S*-enantiomers. Overall, the deleterious health effects of EAs are associated with the ability of EAs to act as ligands for a variety of receptors. The ligand activity is reported to be greater for *R*-enantiomers, compared to *S*-enantiomers [18,20].

The fractionation of EAs amongst milling products and the epimerization observed during milling and cooking highlights the need for exposure assessments to consider concentrations in food products, or to use a processing factor, as opposed to using whole grain durum EA concentrations to estimate consumers’ exposure. The results of this study also indicate that both *R*- and *S*-enantiomers should be monitored and assessed, in order to obtain an accurate view of consumers’ exposure to EAs.

## 4. Materials and Methods

### 4.1. Samples

A No.1 Canada Western Amber Durum sample (CWAD, 30 kg) was cleaned and hand-picked to remove ergot bodies and any kernels with dark discoloration. The cleaned CWAD wheat that did not contain ergot sclerotia was divided into 6 × 5 kg sub-samples. Sclerotia hand-picked out of other naturally-infected CWAD samples were added back to five of the sub-samples in order to prepare grain with 0.01, 0.02, 0.03, 0.04, and 0.1% (m/m) ergot sclerotia. The sixth 5 kg sub-sample was kept at 0% ergot sclerotia. The 5 kg sub-samples were divided into 2 × 2 kg and 1 × 1 kg portions using a rotary sample divider (Materials Sampling Solutions, Southport, Australia). The 2 × 2 kg portions were milled as described below. The 1 kg whole grain portion was comminuted using a Retsch SR 300 rotor beater mill fitted with a 750 μm screen and coupled with a Retsch DR 100 vibratory feeder.

### 4.2. Milling and Processing into Spaghetti

Durum wheat samples were milled on a four stand Allis-Chalmers laboratory mill coupled with a small-scale semolina purifier previously described by Dexter et al. [22]. The mill flow consists of four corrugated break roll passages, five corrugated sizing roll passages, and 10 purification steps. The mill room was controlled at 21 °C and 60% relative humidity. Durum wheat samples were tempered to 16% moisture for 16 h before milling. Particles retained on a 425 μm screen after the four break passages were collected as bran. After each break passage, coarse material retained on a 630 μm screen was passed through sizing rolls. After sizing passages, flour was collected by combining materials with fine particles passing through the 180 μm sieve, and particles retained on a 700 μm screen were collected as shorts. The remaining milling streams were passed through a purifier. After each purification step, streams with particles passing through the 571 μm screens, but retained on the 183 μm screens, were collected as semolina. Particles retained on the 630 μm screens on purifiers 7–10 were collected as feed.

Following the method of Fu et al. [23], spaghetti was produced from semolina using a customized micro-extruder (Randcastle Extrusion Systems Inc., Cedar Grove, NJ, USA). For dough preparation, semolina was mixed with water in a high speed asymmetric centrifugal mixer (DAC 400 FVZ SpeedMixer, FlackTec, Landum, SC, USA) at constant water absorption of 31.5%. Vacuum was applied to eliminate the introduction of air bubbles, after which the dough crumbs were extruded through a four-hole Teflon coated spaghetti die (1.8 mm). 

The fresh pasta was subsequently dried in a pilot pasta dryer (Bühler, Uzwil, Switzerland) coupled with a 325 min drying cycle and maximum temperature of 85 °C.

### 4.3. Cooking and Moisture Content Determination

Spaghetti (10 g) was added to 100 g of water brought to boil. The spaghetti was cooked uncovered for 10 min. After 10 min, cooked spaghetti was immediately removed from the cooking water with tongs, excess adhered water was left to evaporate for 1 min, and the mass of cooked spaghetti was determined. A 10 g portion of boiled spaghetti was then placed into a polyethylene sample bottle, which was closed and stored for approximately 2 h until extraction and EA analysis. The remainder of the boiled spaghetti was re-weighed, stored in sealed plastic bags, and frozen until moisture content determination. The remaining cooking water was cooled in a refrigerator and stored for approximately 4 h until extraction and EA analysis.

The moisture content of cooked spaghetti was determined gravimetrically. The remainders of the boiled spaghetti that were weighed immediately after cooking were placed on sieves and air dried for 24 h. The air dried samples were ground using a Brabender MLI-204 break mill, and then dried for 1 h in an oven held at 130 °C. The moisture content of the cooked spaghetti was calculated from the decrease between the post-cooking and air dried masses as well as the pre- and post-oven dried masses.

Moisture content of freshly extruded spaghetti was calculated based on the amount of water added and originally present in the semolina, minus 0.5% during to moisture loss during pasta processing.

### 4.4. Determination of Ergot Alkaloids

Samples were analyzed for 10 ergot alkaloids according to the liquid chromatography tandem mass spectrometry method described by Tittlemier et al. [5]. The 10 ergot alkaloid analytes are listed in Table 2. Four of the alkaloids were *S*-epimers of *R*-enantiomers. The two *S*-epimers that were not included in the method (ergonovinine and ergotaminine) were not easily obtained at the time of analysis. In order to correct for variations in final extract volume and injection volume, dihydroergotamine was used as an internal standard.

Milling products and spaghetti (10 g) were extracted with 50 mL 84:16 (*v*/*v*) acetonitrile/3.03 mM aqueous ammonium carbonate. The slurries of milling products were shaken on a flatbed shaker for 30 min, and spaghetti was comminuted in extraction solvent using a handheld laboratory homogenizer for 3 min at 12,000 rpm. After extraction, sample extracts were then centrifuged, and an aliquot of supernatant was diluted with 3.03 mM aqueous ammonium carbonate. Internal standard was added to all samples prior to analysis. Post-extraction fortified calibration standards were prepared in wheat matrix.

Water (1 g) was transferred from the cooled cooking water, combined with 1 mL acetonitrile, and vortex mixed for 10 s. The solution was left to sit for 2 min prior to a 1 mL aliquot being taken and combined with 1.5 mL of 3.03 mM aqueous ammonium carbonate. Internal standard was added, final sample extracts were then vortex mixed and filtered using PTFE filters prior to analysis.

Sample extracts were chromatographed on a C_18_ column followed by analysis in the positive electrospray ionization mode using multiple reaction monitoring. Two transitions were monitored for each analyte. In order to avoid epimerization of ergot alkaloids, the exposure of sample extracts to light was minimized by covering samples and extracts during processing, and by keeping the illumination option off on the liquid chromatograph sample manager.

Analytes were considered to be positively identified and quantitated if their retention times were within 0.1 min of the average retention time of the corresponding analyte in the external calibration standards; the peak had a signal-to-noise ratio greater than 9:1, and the ratio of qualification to quantitation ions was within acceptable tolerances [24]. Analyte peak areas were normalized to the dihydroergotamine peak area in samples during calculation of concentrations.

Blank wheat samples fortified with a solution of standards and an in-house rye reference material were analyzed with each batch of samples and were used to monitor the performance of the analytical method during the study.

### 4.5. Evaluation of Method for Analysis of Cooking Water and Boiled Spaghetti

Because water and boiled spaghetti were different matrices than the grain used in the initial validation of the EA analytical method [5], additional method evaluation was performed for these two matrices. Boiled commercially-available durum spaghetti, and the cooking water used to boil the spaghetti, were fortified with ergot alkaloids, and analyzed as described above.

The boiled spaghetti was fortified with the *R*-enantiomers only; fortification concentrations were 200 μg/kg for each alkaloid aside from ergonovine, which was fortified at 40 μg/kg. The boiled spaghetti was drained, and as much water adhering to the cooked pasta as possible was removed by shaking. The cooked spaghetti was then weighed within a minute after boiling to obtain a relevant cooked mass, and 10 g was transferred to a 250 mL centrifuge bottle, capped, and stored in darkness for 30 min to cool prior to fortification. After fortification, samples sat for 20 min in darkness at room temperature prior to extraction. Extraction and analysis was performed as described above, using a ratio of 5:1 (*v*/*m*) extraction solvent to mass of boiled spaghetti.

The cooking water was fortified with both *R*- and *S*-enantiomers. After the cooked spaghetti was removed, the water was placed in a refrigerator for 30 min. After cooling, two portions of 10 mL were removed. One portion was fortified with *R*-enantiomers, and the other was fortified with *S*-enantiomers. Concentrations of ergot alkaloids in both portions were as mentioned above for boiled spaghetti. Three aliquots were taken from the *R*-enantiomers fortified 10 mL, and two aliquots were taken from the *S*-enantiomers fortified 10 mL. Extraction and analysis was performed as described above. All aliquots were analyzed for all ergot alkaloids in order to examine if epimerization had occurred during sample processing.

### 4.6. Statistical Analyses

Statistical analyses were performed using SigmaPlot 13.0 (Systat Software Inc., Chicago, IL, USA). The mean molar percentages of ergocristine and ergocristinine were compared amongst milling products using a Kruskal-Wallis One Way Analysis of Variance on Ranks. The Tukey Test was used to isolate milling products whose mean molar percentages differed from others. Statistical tests were limited to these two EAs because they were the predominant compounds in the milling products.

## Figures and Tables

**Table 1:** Total ergot alkaloids concentration (μg/kg) in whole grain durum and milling products. Average ± standard deviation (2 × 2 kg mill runs for each of the 6 samples) milling yields are provided for the milling products.

	Whole Grain	Bran	Shorts	Feeds	Semolina 1	Semolina 2	Semolina 3	Flour	3rd Break Flour
Milling yield (%, m/m)		12.4 ± 0.1	5.5 ± 0.1	5.3 ± 0.1	61.3 ± 0.1	5.2 ± 0.1	2.39 ± 0.04	3.49 ± 0.07	4.46 ± 0.05
No ERG	<2	<2	<2	12	16	<2	<2	<2	<2
ERG 0.01%	446	1112	241	946	23	337	406	177	188
ERG 0.02%	601	1392	2267	2231	173	368	836	376	313
ERG 0.03%	681	2996	2298	3173	186	461	1005	506	412
ERG 0.04%	936	5715	3482	4532	279	1358	1300	618	530
ERG 0.1%	2147	5695	8002	9955	579	1603	3750	1493	1165

**Table 2:** Mean ± standard deviation percent recovery of ergot alkaloids from cooking water and cooked spaghetti. Cooking water samples were fortified with ergot alkaloids to produce a concentration of 40 μg/kg for ergonovine, 200 μg/kg for the other *R*-enantiomers and 100 μg/kg for the *S*-enantiomers. Boiled spaghetti samples were fortified to produce a concentration of 40 μg/kg for ergonovine and 200 μg/kg for the other *R*-enantiomers; spaghetti was not fortified with *S*-enantiomers. Triplicate replicates of cooking water and boiled spaghetti were analyzed for *R*-isomers. Duplicate replicates of cooking water were analyzed for *S*-isomers.

	Cooking Water	Boiled Spaghetti
Ergonovine	102 ± 3	143 ± 5
Ergosine	97 ± 5	102 ± 1
Ergotamine	100 ± 1	102 ± 1
Ergocornine	103 ± 1	98 ± 1
Ergocryptine	103 ± 4	100 ± 1
Ergocristine	113 ± 2	143 ± 5
Ergosinine	98 ± 9	-
Ergocorninine	93 ± 7	-
Ergocryptinine	104 ± 9	-
Ergocristinine	112 ± 11	-

**Table 3:** Mean ± standard deviation total ergot alkaloids concentration (μg/kg) in freshly extruded spaghetti, cooked spaghetti, and remaining cooking water.

	Freshly Extruded Spaghetti ^1^	Cooked Spaghetti ^2^	Cooking Water ^2^
Moisture content (%, m/m)	34	66	-
No ERG	<2	<2	<2
ERG 0.01%	42.2 ± 0.9	26	<2
ERG 0.02%	114 ± 12	101	2
ERG 0.03%	143 ± 13	84	2
ERG 0.04%	175 ± 13	124	5
ERG 0.1%	533 ± 13	244	10

^1^*n* = 2; ^2^ *n* = 1.
